# Fatty acids negatively regulate platelet function through formation of noncanonical 15‐lipoxygenase‐derived eicosanoids

**DOI:** 10.1002/prp2.1056

**Published:** 2023-01-28

**Authors:** Adriana Yamaguchi, Christopher van Hoorebeke, Benjamin E. Tourdot, Steven C. Perry, Grace Lee, Nicole Rhoads, Andrew Rickenberg, Abigail R. Green, James Sorrentino, Jennifer Yeung, J. Cody Freedman, Theodore R. Holman, Michael Holinstat

**Affiliations:** ^1^ Department of Pharmacology University of Michigan Ann Arbor Michigan USA; ^2^ Department of Chemistry and Biochemistry University of California Santa Cruz Santa Cruz California USA; ^3^ Department of Internal Medicine, Division of Cardiovascular Medicine University of Michigan Ann Arbor Michigan USA

**Keywords:** 15‐lipoxygenase, lipoxygenase, platelet

## Abstract

The antiplatelet effect of polyunsaturated fatty acids is primarily attributed to its metabolism to bioactive metabolites by oxygenases, such as lipoxygenases (LOX). Platelets have demonstrated the ability to generate 15‐LOX‐derived metabolites (15‐oxylipins); however, whether 15‐LOX is in the platelet or is required for the formation of 15‐oxylipins remains unclear. This study seeks to elucidate whether 15‐LOX is required for the formation of 15‐oxylipins in the platelet and determine their mechanistic effects on platelet reactivity. In this study, 15‐HETrE, 15‐HETE, and 15‐HEPE attenuated collagen‐induced platelet aggregation, and 15‐HETrE inhibited platelet aggregation induced by different agonists. The observed anti‐aggregatory effect was due to the inhibition of intracellular signaling including αIIbβ3 and protein kinase C activities, calcium mobilization, and granule secretion. While 15‐HETrE inhibited platelets partially through activation of peroxisome proliferator‐activated receptor β (PPARβ), 15‐HETE also inhibited platelets partially through activation of PPARα. 15‐HETrE, 15‐HETE, or 15‐HEPE inhibited 12‐LOX in vitro, with arachidonic acid as the substrate. Additionally, a 15‐oxylipin‐dependent attenuation of 12‐HETE level was observed in platelets following ex vivo treatment with 15‐HETrE, 15‐HETE, or 15‐HEPE. Platelets treated with DGLA formed 15‐HETrE and collagen‐induced platelet aggregation was attenuated only in the presence of ML355 or aspirin, but not in the presence of 15‐LOX‐1 or 15‐LOX‐2 inhibitors. Expression of 15‐LOX‐1, but not 15‐LOX‐2, was decreased in leukocyte‐depleted platelets compared to non‐depleted platelets. Taken together, these findings suggest that 15‐oxylipins regulate platelet reactivity; however, platelet expression of 15‐LOX‐1 is low, suggesting that 15‐oxylipins may be formed in the platelet through a 15‐LOX‐independent pathway.

## Graphical abstract

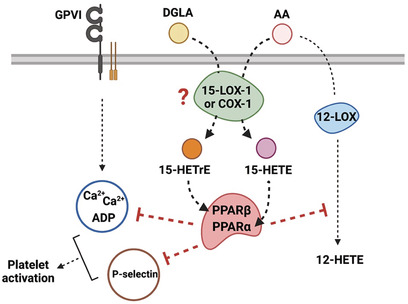

**Abbreviations**
AA: arachidonic acid
ASA: aspirin
COX: cyclooxygenase
CVX: convulxin
d_4_‐13HODE: 13S‐hydroxy‐9Z,11E‐octadecadienoic‐9,10,12,13‐d_4_ acid
DGLA: dihomo‐γ‐linolenic acid
DMSO: dimethyl sulfoxide
DPA: docosapentaenoic acid
EPA: eicosapentaenoic acid
GAPDH: glyceraldehyde 3‐phosphate dehydrogenase
LOX: lipoxygenase
NSAIDS: non‐steroidal anti‐inflammatory drug
PKC: protein kinase C
PMNs: polymorphonuclear leukocytes
PPAR: peroxisome proliferator‐activated receptor
PUFA: polyunsaturated fatty acid
TXA_2_
: thromboxane A_2_
TXB_2_
: thromboxane B_2_
VASP: vasodilator‐stimulated phosphoprotein
7S‐HDHA: 7(S)‐hydroxydocosahexaenoic acid
7S‐HpDHA: 7(S)‐hydroperoxydocosahexaenoic acid
8,15‐diHETE: 8,15‐dihydroxyeicosatetraenoic acid
12‐HETE: 12(S)‐hydroxyeicosatetraenoic acid
12‐HETrE: 12(S)‐hydroxyeicosatetrienoic acid
14‐HpDHA: 14(S)‐hydroperoxydocosahexaenoic acid
14,15‐diHETE: 14,15‐dihydroxyeicosatetraenoic acid
15‐LOX: 15‐lipoxygenase
15‐HEPE: 15(S)‐hydroxyeicosapentaenoic acid
15‐HpEPE: 15(S)‐hydroperoxyeicosapentaenoic acid
15‐HETE: 15(S)‐hydroxyeicosatetraenoic acid
15‐HpETE: 15(S)‐hydroperoxyeicosatetraenoic acid
15‐HETrE: 15(S)‐hydroxyeicosatrienoic acid
15‐HpETrE: 15(S)‐hydroperoxyeicosatrienoic acid
19‐HETE: 19(S)‐hydroxyeicosatetraenoic acid

**Significance Statement**

We demonstrate that in vitro treatment with 15‐HETrE, 15‐HETE, or 15‐HEPE negatively regulates platelet reactivity through inhibition of platelet intracellular signaling that attenuates platelet activation and reduces 12‐LOX activity. These effects are mediated, in part, through the activation of PPARs.

## 1 INTRODUCTION

Long‐chain polyunsaturated fatty acids (PUFAs) are shown to be protective against cardiovascular diseases^1^, ^2^; however, the mechanism of this effect is not well understood. PUFAs have been shown to regulate and alter platelet function through their metabolism to bioactive oxylipins by the two main oxygenases, cyclooxygenase (COX) and lipoxygenase (LOX).^3^ Platelets express COX‐1, whose inhibition by non‐steroidal anti‐inflammatory drugs (NSAIDS) is thought to be a primary reason for the observed decrease in platelet reactivity.^2^, ^4^ Regarding the role of lipoxygenases in platelets, 12‐LOX is highly expressed and plays an important role in regulating platelet activation.^5^, ^6^ However, the presence of 15‐LOX in the platelet and whether it is required for the formation of 15‐LOX‐derived oxylipins (15‐oxylipins) remains unclear.

Although platelets have been demonstrated to generate 15‐oxylipins, such as 15(S)‐hydroxyeicosatetraenoic acid (15‐HETE) from arachidonic acid (AA) and 15(S)‐hydroxyeicosatrienoic acid (15‐HETrE) from dihomo‐γ‐linolenic acid (DGLA), the source of these molecules is poorly defined.^7^, ^8^ Lipoxygenases could potentially generate these molecules; however, studies have indicated that these 15‐oxylipins are generated by COX.^2^, ^9^ Interestingly, the role of these 15‐oxylipins in platelet biology is also controversial. While 15‐HETrE, 15‐HETE, and 15(S)‐hydroxyeicosapentaenoic (15‐HEPE), from eicosapentaenoic acid (EPA), have been shown to inhibit platelet reactivity,^9^, ^10^ other studies have observed a pro‐aggregatory effect of 15‐HETE on platelet function.^11^, ^12^ With respect to their mechanism of action, oxylipins can inhibit platelet function by increasing cyclic adenosine monophosphate (cAMP) levels via Gα_s_‐coupled receptors, or binding intracellular nuclear receptor, such as peroxisome proliferator‐activated receptors (PPARs).^1^, ^2^ Although 15‐HETE has been reported to interact with PPARs in other cell types (^13^; Setty et al., 1986), the mechanism underlying the effects of 15‐HETE and 15‐HETrE on platelet reactivity is not well understood. Given these contradictory and poorly defined results, a better understanding of the 15‐oxylipins effects on platelet activity is warranted. This study helps to elucidate the mechanism of 15‐oxylipin formation in platelets and determine the effects of 15‐HETrE, 15‐HETE, and 15‐HEPE on the regulation of platelet reactivity.

## 2 METHODS

### 2.1 Isolation of human platelets

All research involving human subjects was carried out in accordance with the Declaration of Helsinki and approved by the University of Michigan Institutional Review Board. Prior to blood collection, written informed consent was obtained from all subjects in this study. Blood was collected into vacutainers containing sodium citrate (Becton, Dickinson and Company [BD]) and centrifuged for 10 min at 200 *g* to obtain platelet‐rich plasma. Acid citrate dextrose (2.5% sodium citrate tribasic, 1.5% citric acid, 2.0% d‐glucose) and apyrase (0.02 U/mL) were added to the platelet‐rich plasma, which was then centrifuged for 10 min at 2000 g. Platelets were resuspended in Tyrode's buffer (10 mM HEPES, 12 mM NaHCO_3_, 127 mM NaCl, 5 mM KCl, 0.5 mM NaH2PO_4_, 1 mM MgCl_2_, and 5 mM glucose) and adjusted to the concentrations described below.

### 2.2 Leukocyte depletion of platelets

Washed human platelets (5 × 10^8^ platelets/mL) were incubated with magnetic CD45 MicroBeads (10 μL/mL) (Miltenyi Biotec Inc.) for 30 min. Following incubation, platelets were treated with EDTA (2.5 mM) and filtered through a magnetic‐activated cell sorting separation column that selectively captured CD45‐positive cells. Platelets were pelleted from the column flow‐through by centrifugation following treatment with acid citrate dextrose and apyrase, as described above.

### 2.3 Quantification of platelet‐derived 15‐oxylipins

Washed human leukocyte‐depleted platelets (3 × 10^8^ platelets/mL) were incubated with 10 μM DGLA (dimethyl sulfoxide (DMSO) as control) for 10 min at 37°C, pelleted at 1000 g for 1 min, and the supernatant was frozen. Subsequently, 13S‐hydroxy‐9Z,11E‐octadecadienoic‐9,10,12,13‐d_4_ acid (d_4_‐13HODE) (20 ng) was added to the thawed supernatant and oxylipins were extracted with 1.5 mL dichloromethane, reduced with 20 μL of trimethyl phosphite, and dried under a stream of N_2_. Samples were resuspended in 50 μL of methanol (MeOH) containing 10 ng of d_8_‐12HETE. Prior to chromatography, 100 μL of 0.1% formic acid in water was added to samples, and 90 μl was injected for analysis. UPLC‐MS/MS was performed to monitor the oxylipin production, as previously described,^14^ with the addition of the following m/z transitions: 15‐HETE: 319→219, 15‐HETrE: 321→221, 14,15‐diHETE: 335→205, 8,15‐diHETE: 335→155. Quantitation was performed with a 15‐HETE standard curve. Quantitation of 14,15‐diHETE was based on the relative ionization efficiency of it to 15‐HETE as the standard (0.98 ± 0.2).

### 2.4 Production and isolation of 15‐oxylipins

The synthesis of 15‐HETrE, 15‐HETE, and 15‐HEPE was performed as previously described.^15^, ^16^ Briefly, 15S‐hydroperoxy‐8Z,11Z,13E‐eicosatrienoic acid (15‐HpETrE), 15S‐hydroperoxy‐5Z,8Z,11Z,13E‐eicosatetraenoic acid (15‐HpETE), and 15S‐hydroperoxy‐5Z,8Z,11Z,13E,17Z‐eicosapentaenoic acid (15‐HpEPE) were synthesized by reaction of DGLA, AA, or EPA, respectively, (25‐50 μM) with soybean lipoxygenase‐1. The hydroperoxide products, 15‐HpETrE, 15‐HpETE, and 15‐HpEPE were reduced to the alcohols, 15‐HETrE, 15‐HETE, and 15‐HEPE with trimethyl phosphite. The 15‐oxylipins were then purified by HPLC using a C18 HAISIL 250 × 10 mm semiprep column isocratically in a mobile phase containing 54.5:44.5:1 mixture of acetonitrile, water, and formic acid, respectively.

### 2.5 Platelet aggregation and dense granule secretion

A Chrono‐log Model 700D Lumi‐aggregometer was used to measure platelet aggregation and ATP release in washed human platelets (3 × 10^8^ platelets/mL) under stirring conditions (1100 rpm) at 37°C for 6 min, following the addition of collagen (Chrono‐log), thrombin (Enzyme Research Laboratories), AA (Cayman Chemical Company), or adenosine diphosphate (ADP) (Sigma‐Aldrich).

### 2.6 Protein kinase C substrate phosphorylation

Washed human platelets (3 × 10^8^ platelets/mL) were incubated with oxylipin prior to stimulation with collagen for 5 min in an aggregometer. Reactions were stopped with the addition of 5X Laemmli sample buffer (Tris 1.5 M, pH 6.8; 10% sodium dodecyl sulfate, 50% glycerol, 25% β‐mercaptoethanol, 0.6% bromophenol blue), boiled, and separated on an SDS‐PAGE gel. Western blots were performed with antibodies to GAPDH **(**Santa Cruz Biotechnology) and protein kinase C (PKC) substrate (Cell Signaling Technology).

### 2.7 Quantification of calcium mobilization and αIIbβ3 activation via flow cytometry

Washed human platelets (1 × 10^6^ platelets/mL) were incubated with DMSO, 15‐HETrE, or 15‐HETE (10 μM) for 10 min at 37°C. Platelets were then treated for 5 min with either Fluo‐4‐AM (0.5 μg; Thermo Fisher Scientific) or PAC‐1 (BD Pharmingen), an antibody that binds the active conformation of αIIbβ3. Platelets, supplemented with CaCl_2_ (1 mM), were stimulated with convulxin (2.5 ng/mL, purchased from Dr. Kenneth J. Clemetson, Theodor Kocher Institute, University of Berne, Bern, Switzerland) and the mean fluorescence intensity of the sample was continuously measured on an Accuri C6 flow cytometer (BD Biosciences).

### 2.8 α‐granule secretion

Washed human platelets (3 × 10^8^ platelets/mL) treated with oxylipin for 10 min were stimulated with collagen (5 μg/mL) for 5 min under stirring conditions in the presence of the tetrapeptide Arg‐Gly‐Asp‐Ser (RGDS; 2 mM; Sigma‐Aldrich) to prevent platelet aggregation. A PE‐conjugated P‐selectin antibody (BD Pharmingen) was added to the stimulated platelets for 10 min and P‐selectin surface expression was quantified by flow cytometry.

### 2.9 Vasodilator‐stimulated phosphoprotein phosphorylation

Washed human platelets (3 × 10^8^ platelets/mL) were incubated with oxylipins (10 μM), forskolin (5 μM), or DMSO for 10 min prior to the addition of 5X Laemmli sample buffer. Samples were boiled and then separated on a SDS‐PAGE gel. Western blots were performed with antibodies to phosphorylated (pS157) and total vasodilator‐stimulated phosphoprotein (VASP) **(**Santa Cruz Biotechnology).

### 2.10 15‐LOX‐1 and 15‐LOX‐2 expression in platelets

The expression of 15‐LOX‐1 and 15‐LOX‐2 was assessed in washed human leukocyte‐depleted platelets (3 × 10^8^ platelets/mL) and non‐depleted platelets (3 × 10^8^ platelets/mL). The purified 15‐LOX‐1 (3.7 μg/lane), 15‐LOX‐2 (7.4 μg/lane), and 12‐LOX (7.5 μg/lane) enzymes were used as control. 5X Laemmli sample buffer was added to the platelets, and samples were boiled and then separated on a SDS‐PAGE gel. Western blots were performed with antibodies to 15‐LOX‐1 (Abcam) or 15‐LOX‐2 (Abcam), and β‐actin (Cell Signaling Technology).

### 2.11 Thromboxane B_2_ (TXB_2_ ) and 12‐HETE formation

Washed human platelets (3 × 10^8^ platelets/mL) pretreated with 15‐HETrE, 15‐HETE, or 15‐HEPE (10 μM) were stimulated with collagen (5 μg/mL) for 5 min in an aggregometer. Platelets were pelleted by centrifugation at 1000 g for 1 min and the supernatant was transferred to a new tube. The supernatant was immediately placed on dry ice. Thromboxane B_2_ (TXB_2_) and 12(S)‐hydroxyeicosatetraenoic acid (12‐HETE) were quantified by UPLC‐MS/MS, as described above.

### 2.12 Mass spectrometry analysis of 12‐LOX enzymatic products from 15‐oxylipin substrates

Briefly, 12‐LOX (60 pmoles) was reacted with 10 μM of 15‐HETE, 15‐HETrE, and 15‐ HEPE separately at 25°C, quenched after completion of the reaction, extracted three times with dichloromethane, reduced with trimethyl phosphite, and evaporated under a stream of nitrogen gas. Reactions were analyzed via LC‐MS/MS. The chromatography system was coupled to a Thermo‐Electron LTQ LC‐MS/MS for mass analysis. All analyses were performed in negative ionization mode at the normal resolution setting. MS^2^ was performed in a targeted manner with a mass list containing the following m/z ratios ±0.5: 317.5 (HEPEs), 319.5 (HETEs), 321.5 (HETrEs), 331.5 (diHEPEs), 335.5 (diHETEs), 337.4 (diHETrEs), 349.5 (triHEPEs (349.5), 351.5 (triHETEs), and 353.5 (triHETrEs).

### 2.13 Kinetic analysis of AA, DGLA, EPA, and corresponding 15‐oxylipins with 12‐LOX

Overexpression and purification of human 12‐LOX were performed as previously described.^17^ 12‐LOX steady‐state kinetic reactions were constantly stirred at ambient temperature, in a 1 cm^2^ quartz cuvette containing 2 ml of 25 mM HEPES, pH 8 with DGLA, AA, EPA, 15‐HpETrE, 15‐HpETE, or 15‐HpEPE. Substrate concentrations were varied from 0.25 to 10 μM for the PUFA reactions or 0.5 to 25 μM for the 15‐oxylipin reactions. Concentrations of PUFA were determined by measuring the amount of 15‐oxylipin produced from a complete reaction with soybean lipoxygenase‐1. Concentrations of 5S‐HETE, 5S‐HpETE, 7S‐HDHA, and 7S‐HpDHA were determined by measuring the absorbance at 234 nm. Reactions were initiated by the addition of h15‐LOX‐1 (~20‐60 nmol) and were monitored on a Perkin‐Elmer Lambda 45 UV/VIS spectrophotometer. Product formation was determined by the increase in absorbance at 234 nm for 15‐oxylipins (ε_234_ = 27 000 M^−1^ cm^−1^) and 270 nm for di‐oxylipins (ε_270_ = 37 000 M^−1^ cm^−1^).^18^, ^19^ KaleidaGraph (Synergy) was used to fit initial rates, as well as the second‐order derivatives (*k*_cat_ */K*_M_) to the Michaelis‐Menten equation for the calculation of kinetic parameters.

### 2.14 Determination of IC_50_ of 15‐HETrE, 15‐HETE, and 15‐HEPE against 12‐LOX

Purified 12‐LOX (approximately 60 pmol) was added to 10 μM AA in 2 ml of 25 mM HEPES (adjusted up to pH 8.0) at 25°C, in the presence of the oxylipin, with the oxylipin dissolved in methanol. For control rates, the exact conditions were performed with equivalent methanol volumes, minus the oxylipin. IC_50_ values were obtained by determining the initial enzymatic rate at various 15‐HETrE, 15‐HETE, and 15‐HEPE concentrations and plotting the rates against 15‐HETrE, 15‐HETE, and 15‐HEPE concentrations, followed by a hyperbolic saturation curve fit via KaleidaGraph (Synergy).

### 2.15 Statistical analysis

Two‐tailed paired *t* test, one‐way, and two‐way analysis of variance (ANOVA) were performed with Prism 9 (GraphPad Software) to analyze the data. The statistical test used in each assay is reported in the figure legend. Data represent mean values ± standard error of the mean (SEM) or mean values ± standard deviation (SD), as reported in the figure legends.

## 3 RESULTS

### 3.1 The 12‐HETrE/15‐HETrE ratio in platelets treated with DGLA

**FIGURE 1:**
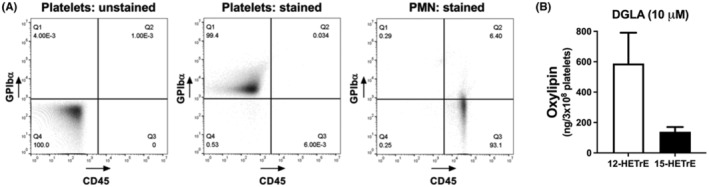
Leukocyte‐depleted platelets produce 15‐HETrE. (A) Leukocyte‐depleted platelets were stained with antibodies specific to platelets (GPIbα) and leukocytes (CD45) and analyzed by flow cytometry to quantify the number of residual polymorphonuclear leukocytes (PMNs) (CD45‐positive, GPIbα‐negative cells) in each sample. (B) The levels of 12‐HETrE and 15‐HETrE were measured in the releasate of leukocyte‐depleted platelets (*n* = 3) treated with DGLA (10  μM). Data represent mean ± SD.

12‐LOX is highly expressed in platelets^5^, ^6^ and our laboratory has already demonstrated that supplementation of platelets with DGLA increased the formation of 12‐HETrE, a 12‐LOX‐derived oxylipin.^20^ In order to determine whether platelets were able to form 15‐oxylipins, the levels of 12‐HETrE and 15‐HETrE in the releasate of platelets treated with DGLA (10 μM) were quantified by mass spectrometry to determine their ratio of formation from a common sample. Since leukocytes are a common contaminate of isolated platelets and a potential source of 15‐HETrE, washed human platelets were leukocyte depleted by magnetic‐activated cell sorting (^21^, ^22^). The purity of the leukocyte‐depleted platelets was quantified by flow cytometry using leukocyte (CD45) and platelet (GPIbα)‐specific antibodies. The leukocyte‐depleted platelets contained 40 ± 11.34 (mean ± SEM; *n* = 7) leukocytes per million platelets as detected by flow cytometry (Figure 1A). Leukocyte‐depleted platelets treated with DGLA produced 4.2 times as much 12‐HETrE (588 ± 352 ng/3 × 10^8^ platelets) as 15‐HETrE (139 ± 54 ng/3 × 10^8^ platelets) **(**Figure 1B **)**.

### 3.2 15‐Oxylipins inhibit agonist‐induced platelet aggregation

**FIGURE 2:**
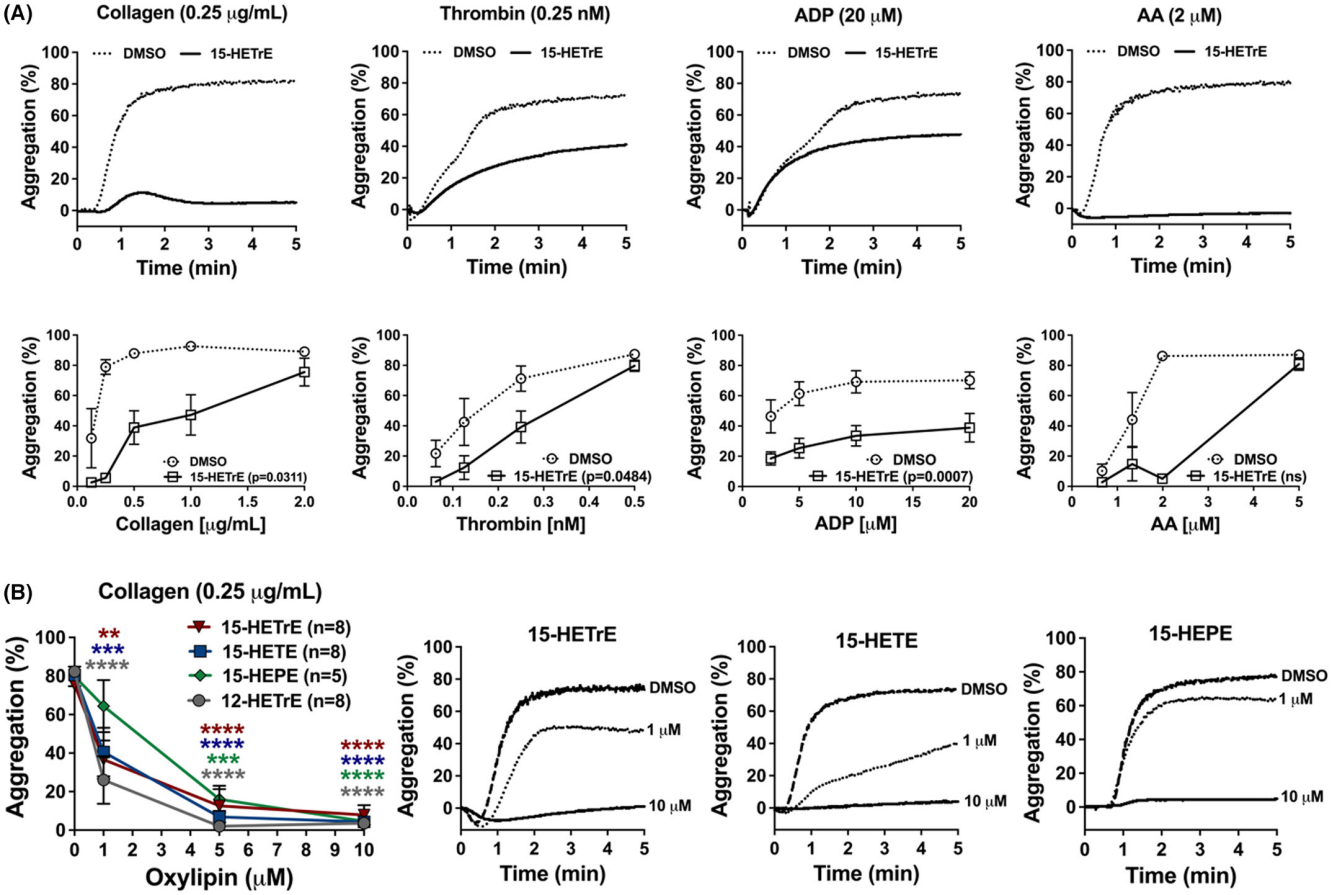
15‐HETrE inhibits platelet aggregation in response to different agonists. (A) Washed human platelets were treated with 15‐HETrE (10 μM) or vehicle (DMSO) for 10 min and then stimulated with increasing concentrations of collagen (*n* = 4–6, *p* = .0311), thrombin (*n* = 6, *p* = .0484), ADP (*n* = 6, *p* = .0007), or AA (*n* = 5‐6, 0.2720) in an aggregometer. Data represent mean ± SEM of the maximum aggregation (bottom panel) and representative data (top panel). One‐way ANOVA statistical analysis with Dunnett's multiple comparison post hoc test. (B) Washed human platelets (*n* = 5‐8) were treated with increasing concentrations of 12‐HETrE, 15‐HETrE, 15‐HETE, or 15‐HEPE and then stimulated with collagen (0.25 μg/mL). Data represent mean ± SEM on the right and representative data are shown in the three graphs on the left. One‐way ANOVA statistical analysis with Dunnett's multiple comparison post hoc test was performed between DMSO and oxylipins. *p < .05, **p < .01, ***p < .001, ****p < .0001. DMSO concentration corresponds to the residual DMSO in the 10 μM oxylipin sample.

We have shown that human platelets treated with DGLA were able to form 15‐HETrE ex vivo (Figure 1B). While micromolar levels of 15‐HETrE were previously reported to inhibit thromboxane receptor‐mediated platelet aggregation,^9^ it remained unknown whether 15‐HETrE inhibited platelet activation through other receptors such as PPARs, GPVI, or P2Y_12_. In order to determine the efficacy of 15‐oxylipins at inhibiting platelet aggregation in response to different platelet agonists, aggregation was measured in 15‐HETrE‐treated platelets stimulated with increasing concentrations of collagen, thrombin, ADP, or AA. 15‐HETrE was effective at attenuating aggregation in response to collagen, thrombin, and ADP, but not AA. However, higher doses of the agonists (collagen or thrombin) were able to overcome the inhibitory effects of 15‐HETrE (Figure 2A).

Following the evaluation of the effects of 15‐HETrE on aggregation in response to different agonists, we decided to determine whether other 15‐oxylipins derived from two major PUFA precursors in the plasma membrane have an effect on platelet aggregation. The effects of the oxylipins 15‐HETE from AA and 15‐HEPE from EPA on platelet aggregation were assessed in response to collagen, a potent endogenous agonists known to directly activate platelets.Washed human platelets were treated with increasing concentrations of 15‐HETrE, 15‐HETE, or 15‐HEPE prior to stimulation with collagen, the GPVI and α2β1 agonist, to determine whether the 15‐oxylipins inhibited collagen‐mediated platelet aggregation. 15‐HETrE, 15‐HETE, and 15‐HEPE all inhibited platelet aggregation in response to collagen (0.25 μg/mL), with maximal inhibition of aggregation achieved at 10 μM (Figure 2B). Collagen‐stimulated platelets were also incubated with 12(S)‐hydroxyeicosatetrienoic acid (12‐HETrE) to determine the relative potency of 15‐oxylipins‐dependent inhibition of aggregation compared to previously identified antiplatelet monohydroxylated oxylipins.^14^, ^20^, ^23^, ^24^ The three monohydroxylated 15‐oxylipins (15‐HETrE, 15‐HETE, or 15‐HEPE) inhibited collagen‐mediated platelet aggregation with similar potency to each other and to that of 12‐HETrE (Figure 2B).

### 3.3 12‐LOX product profile and kinetics with C20 PUFAs and 15‐oxylipins substrates

The biosynthesis pathway of the 15‐oxylipins involves a hydroperoxide precursor, such as 15‐HpTrE for 15‐HETrE, 15‐HpEPE for 15‐HEPE, or 15‐HpETE for 15‐HETE. It is known that the hydroperoxide precursors have the ability to be converted into other oxylipins, which might also have an effect on platelet reactivity. Previously, 15‐HpETE was shown to be a poor substrate for 12‐LOX that was converted into 8,15‐dihydroxy‐eicosatetraenoic acid (8,15‐diHETE) and 14,15‐dihydroxy‐eicosatetraenoic acid (14,15‐diHETE),^8^, ^25^ with 8,15‐diHETE being capable of inhibiting ADP‐induced platelet aggregation.^26^ In this study, we demonstrate that 12‐LOX also poorly converts 15‐HpETrE and 15‐HpEPE to their 8,15‐ and 14,15‐products, with only ~1% being produced before enzymatic inactivation occurs. Of the small amount of di‐oxylipins produced, roughly twice as much of the 14,15‐product was made relative to the 8,15‐product for the three 15‐oxylpins investigated (Table 1). The 8,15‐product is due to the degradation of the 14,15‐epoxide, while the 14,15‐product is due to oxygenation.^27^, ^28^ This mechanism was confirmed by the increase in the 8,15‐product upon lowering the O_2_ concentration (Table 1). It should be noted that the reaction rate of 12‐LOX with the alcohol form of the 15‐oxylipins was significantly reduced relative to the hydroperoxides, consistent with the loss of the formation of the epoxide product, which requires the hydroperoxide substrate.

**TABLE 1:** Product distribution from reacting 12‐LOX with 15‐HpETrE, 15‐HpETE, and 15‐HpEPE.

12‐LOX + substrate	8,15‐product (%)	14,15‐product (%)
15‐HpETrE	77 ± 1	23 ± 1
15‐HpETE	66 ± 2	35 ± 2
15‐HpEPE	70 ± 1	30 ± 2
15‐HpEPE (low O_2_)	83	17

Abbreviations: O_2_, oxygen; 12‐LOX, 12‐lipoxygenase; 15‐HpEPE, 15(S)‐hydroperoxyeicosapentaenoic acid; 15‐HpETE, 15(S)‐hydroperoxyeicosatetraenoic acid; 15‐HpETrE, 15(S)‐hydroperoxyeicosatrienoic acid.

The kinetics of 12‐LOX reacting with the three PUFA substrates, DGLA, AA, and EPA, revealed similar kinetic values (Table 2). The kinetics of the corresponding hydroperoxide 15‐oxylipins of these three PUFAs also revealed similar values (vide supra), indicating that for both the PUFAs and the 15‐oxylipins, the double bond configuration had little effect on kinetics, given that all of these six substrates are 20 carbons in length. However, it should be noted that the kinetic rates of the hydroperoxide 15‐oxylipins were approximately 10‐fold less than that of the PUFA substrates, which agrees with previous results with other oxylipin substrates.^29^, ^30^, ^31^

**TABLE 2:** 12‐LOX kinetics with PUFAs and 15‐oxylipins.

Enzyme + substrate	*k*_cat_ (s^−1^)	*k*_M_ (μM)	*k*_cat_/*k*_M_ (s^−1^ μM^−1^)
DGLA	12 ± 0.3	2.5 ± 0.2	4.9 ± 0.3
AA	11 ± 0.2	0.49 ± 0.07	22 ± 3
EPA	9 ± 0.5	1 ± 0.2	8.7 ± 1.2
15‐HpETrE	0.96 ± 0.05	11 ± 1	0.086 ± 0.004
15‐HpETE	1.5 ± 0.05	5.8 ± 0.4	0.26 ± 0.001
15‐HpEPE	0.93 ± 0.06	9.2 ± 1	0.10 ± 0.008

Abbreviations: AA, arachidonic acid; DGLA, dihomo‐γ‐linolenic acid; EPA, eicosapentaenoic acid; PUFAs, polyunsaturated fatty acids; 12‐LOX, 12‐lipoxygenase; 15‐HpEPE, 15(S)‐hydroperoxyeicosapentaenoic acid; 15‐HpETE, 15(S)‐hydroperoxyeicosatetraenoic acid; 15‐HpETrE, 15(S)‐hydroperoxyeicosatrienoic acid.

### 3.4 12‐LOX allosteric and hypoxic regulation of epoxidation

Oxylipins have been previously determined to dose dependently affect the ratio of di‐oxygenation: epoxidation products,^29^, ^31^ due to allosteric regulation of enzyme mechanism. In order to determine if 15‐HpEPE could also affect the ratio of LOX products, the product profile was assessed in solutions ranging from 1 to 20 μM of 15‐HpEPE. Increased concentrations of 15‐HpEPE reduced epoxide formation from 84% at 1 μM 15‐HpEPE to 58% at 20 μM (Table 3), indicating that 15‐HpEPE is also a 12‐LOX allosteric regulator that affects secondary product formation.

**TABLE 3:** Allosteric effect of 12‐LOX with 15‐HpEPE.

12‐LOX + 15‐HpEPE	1 μM	2 μM	5 μM	10 μM	15 μM	20 μM
% 8,15‐diHEPE	84%	75%	70%	67%	65%	58%

Abbreviations: 12‐LOX, 12‐lipoxygenase; 15‐HpEPE, 15(S)‐hydroperoxyeicosapentaenoic acid; 8,15‐diHEPE, 8,15‐dihydroxy‐eicosatetraenoic acid.

### 3.5 Ex vivo platelet incubation with 15‐oxylipins

The in vitro assays indicate that 12‐LOX reacts slowly with 15‐oxylipins under in vitro conditions and, therefore, the 15‐oxylipins were incubated with platelets in order to determine the reactivity of 12‐LOX under ex vivo conditions. Specifically, the formation of the di‐oxylipins from the 15‐oxylipin was measured in platelets incubated with 15‐HpETE, 15‐HpETrE, or 15‐HpEPE and their reduced alcohol species, all at 10 μM. The first observation is that the total di‐oxylipin produced from all three of the hydroperoxide 15‐oxylipins is comparable, approximately 100 ng per 1 × 10^9^ platelets, and is consistent with the amount of di‐oxylipin produced when 14(S)‐hydroperoxydedocosahexaenoic acid (14‐HpDHA) was added to platelets.^29^ Second, only 1/10 of the reduced alcohol oxylipin products were observed compared to the hydroperoxide oxylipin products, which is consistent with the in vitro kinetics, where the alcohol oxylipins were observed to be poorer substrates than the hydroperoxides. Finally, the 8,15‐product from epoxide degradation was the major di‐oxylipin species when 15‐HpEPE was added to platelets (Table 4), suggesting that the hydroperoxide oxylipin is a viable 12‐LOX substrate in the platelet.

**TABLE 4:** Product distribution from reacting platelets with 15‐HEPE and 15‐HpEPE.

Platelets + substrate	Non‐enzymatic (8,15) product (%)	Dioxygenation (8,15/14,15)‐product (%)
15S‐HEPE	12.6 ± 1.1	87.4 ± 1.1
15S‐HpEPE	75.4 ± 0.8	24.6 ± 0.8

Abbreviations: 15‐HEPE, 15(S)‐hydroxyeicosapentaenoic acid; 15‐HpEPE, 15(S)‐hydroperoxyeicosapentaenoic acid.

### 3.6 Oxylipin inhibition of 12‐LOX activity in both ex vivo and in vitro conditions

**FIGURE 3:**
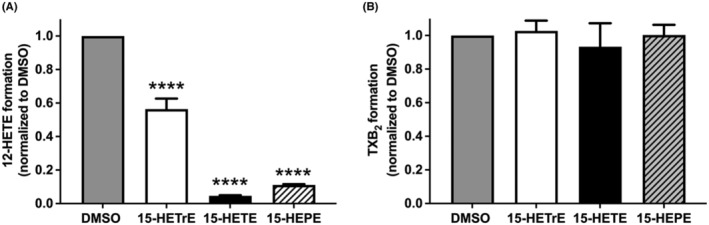
15‐oxylipins inhibit 12‐LOX activity. The levels of (A) 12‐HETE (DMSO = 1.0 ± 0; 15‐HETrE = 0.56 ± 0.139; 15‐HETE = 0.04 ± 0.009; 15‐HEPE = 0.11 ± 0.009) and (B) TXB_2_ (DMSO = 1.0 ± 0; 15‐HETrE = 1.03 ± 0.137; 15‐HETE = 0.93 ± 0.312; 15‐HEPE = 1.0 ± 0.134) were quantified in the lysate of collagen‐stimulated (5 μg/mL) platelets (*n* = 5) pretreated with 15‐ HETE (10 μM), 15‐HETrE (10 μM), or 15‐HETE (10 μM). One‐way ANOVA with Dunnett's multiple comparison post hoc test. Data represent mean ± SD. ****p < 0.0001. DMSO concentration corresponds to the residual DMSO in the 10 μM oxylipin sample.

Previous studies have demonstrated that the antiplatelet effects of 15‐HETE were due, in part, to its ability to selectively inhibit COX,^32^ 12‐LOX,^22^, ^24^, ^33^ or both.^23^ To evaluate whether 15‐HETrE, 15‐HETE, or 15‐HEPE inhibit platelet activation via inhibiting COX or 12‐LOX, the levels of their respective AA‐derived metabolite, TXB_2_ and 12‐HETE, were quantified in the releasate of collagen‐stimulated platelets. Since Ca^2+^ mobilization is required for TXB_2_ and 12‐HETE formation,^34^ a high concentration of collagen (5 μg/mL) was used that caused similar levels of Ca^2+^ mobilization. Platelets incubated with 15‐HETrE (10 μM), prior to collagen stimulation, decreased 12‐HETE formation (0.56 ± 0.139, mean ± SD) by 44 ± 11% (Figure 3A) but had no effect on the formation of TXB_2_ (1.03 ± 0.137, mean ± SD) (Figure 3B), compared to DMSO vehicle‐treated platelets (1.0 ± 0, mean ± SD). Treatment of platelets with 15‐HETE (10 μM) prior to collagen stimulation had a 97 ± 1% decrease in 12‐HETE generation (0.04 ± 0.009, mean ± SD) (Figure 3A), with no effect on TXB_2_ formation (0.93 ± 0.312, mean ± SD) (Figure 3B), compared to vehicle‐treated platelets (1.0 ± 0, mean ± SD). In comparison, platelets incubated with 15‐HEPE (10 μM) prior to collagen stimulation had a 89 ± 1% decrease in 12‐HETE formation (0.11 ± 0.009, mean ± SD) (Figure 3A), with no effect on TXB_2_ formation (1.0 ± 0.134, mean ± SD) (Figure 3B) compared to vehicle‐treated platelets (1.0 ± 0, mean ± SD).

The aforementioned data suggest that treatment of platelets with 15‐HETrE, 15‐HETE, or 15‐HEPE diminished the ability of platelets to produce 12‐HETE ex vivo; however, the mechanism remains unknown. It is possible that these 15‐oxylipins could directly inhibit 12‐LOX. Therefore, the formation of 12‐HETE was measured in vitro following the incubation of AA with recombinant 12‐LOX in the presence of 15‐HETrE (IC_50_ = 105 ± 55 μM), 15‐HETE (IC_50_ = 46 ± 19 μM), or 15‐HEPE (IC_50_ = 142 ± 11 μM) to determine if they directly inhibit 12‐ LOX. However, the low potency of these 15‐oxylipins indicates no direct inhibition of 12‐LOX in the platelet.

### 3.7 15‐Oxylipins inhibit intracellular platelet signaling

**FIGURE 4:**
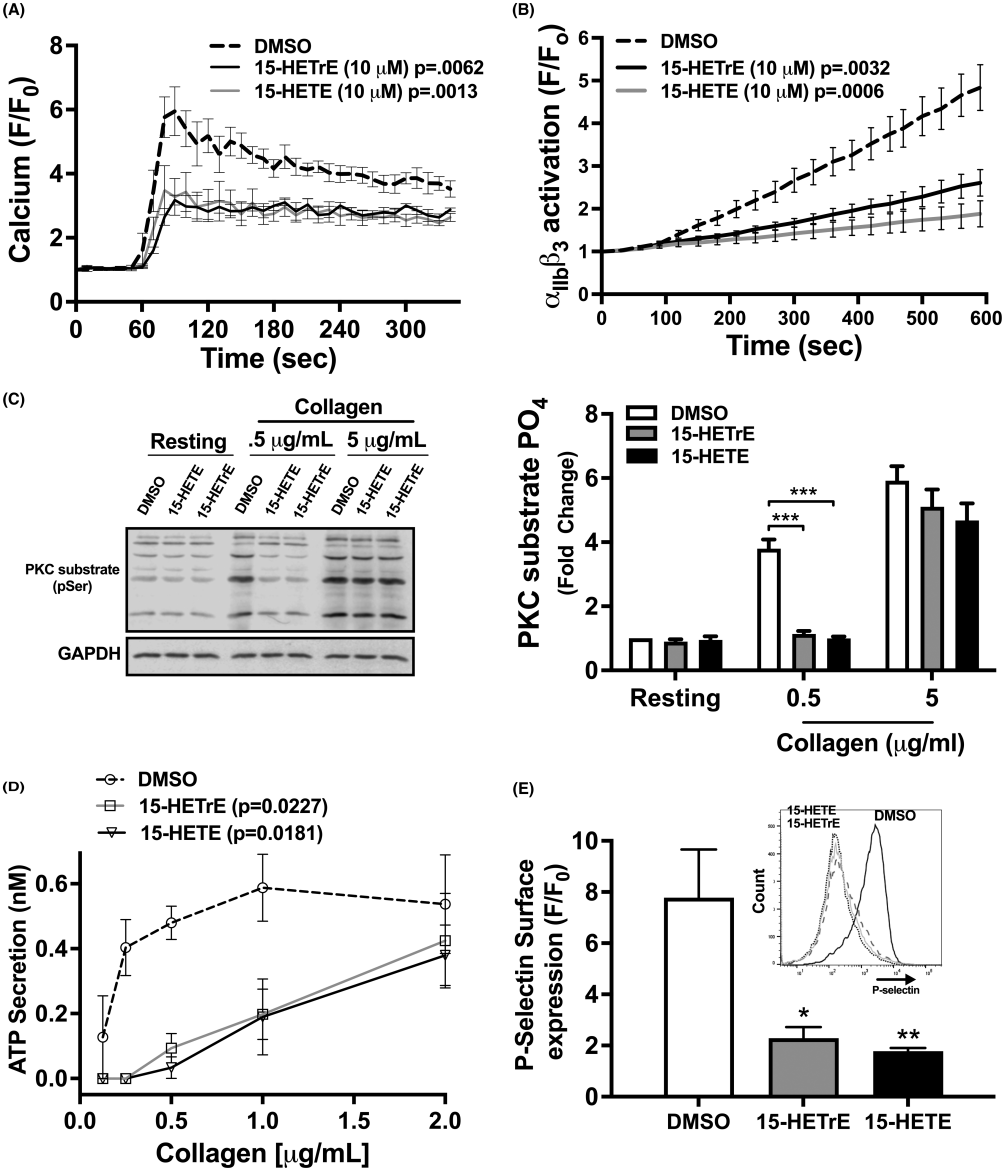
15‐HETE and 15‐HETrE inhibit intracellular platelet signaling. (A) Platelets (*n* = 4) were treated with a 15‐HETE or 15‐HETrE (10 μM) and Fluo‐4‐AM, a cell‐permeable, calcium‐sensitive dye, then stimulated with convulxin (CVX; 2.5 ng/mL), and Ca^2+^ mobilization was analyzed by flow cytometry in real time. (B) Platelets (*n* = 9) that had been treated with 15‐HETE or 15‐HETrE (10 μM) were stimulated with CVX (2.5 ng/mL) in the presence of FITC‐conjugated PAC‐1, an antibody specific to the active conformation of αIIbβ3, and analyzed by flow cytometry in real time. Two‐way ANOVA with Dunnett's multiple comparison post hoc test. (C) Collagen‐stimulated platelets (*n* = 5) pretreated with either 15‐HETE (10 μM) or 15‐HETrE (10 μM) were lysed, and western blots were performed with antibodies to the phospho‐serine PKC substrate motif and GAPDH. Two‐way ANOVA with Dunnett's multiple comparison post hoc test. (D) ATP secretion, a marker of dense granule secretion, was measured from platelets (*n* = 4‐5) incubated with 15‐HETE or 15‐HETrE (10 μM) in a Lumi‐aggregometer in the presence of increasing concentrations of collagen. One‐way ANOVA with Dunnett's multiple comparison post hoc test. (E) P‐selectin surface expression, a marker of α‐granule secretion, was quantified in collagen‐stimulated platelets (*n* = 4) treated with 15‐HETE or 15‐HETrE (10 μM) by flow cytometry using a PE‐conjugated P‐selectin antibody. One‐way ANOVA with Dunnett's multiple comparison post hoc test. Data represent mean ± SEM. *p < 0.05, **p < 0.01, ***p < 0.001. DMSO concentration corresponds to the residual DMSO in the 10 μM oxylipin sample.

The 12‐LOX‐derived oxylipin from AA, 12‐HETE, plays a critical role in enhancing platelet activation,^2^, ^35^ but 12‐HETrE inhibits platelet reactivity and clot formation.^14^, ^36^ Although we have demonstrated that either the 15‐oxylipin from AA (15‐HETE) or DGLA (15‐HETrE) attenuated human platelet aggregation, due to the opposite effect of the 12‐LOX‐derived oxylipin depending on the precursor fatty acid, we focused on elucidating whether different mechanisms were involved in 15‐HETrE or 15‐HETE anti‐aggregatory effects. To assess whether 15‐HETrE or 15‐HETE attenuated platelet aggregation through modulation of intracellular signaling, Ca^2+^ mobilization, integrin activation, PKC activation, and granule secretion were evaluated in GPVI‐stimulated platelets treated with 15‐HETE or 15‐HETrE. Ca^2+^ mobilization, a key regulator of integrin αIIbβ3 activation, was evaluated in platelets treated with oxylipins in real time by flow cytometry to determine if Ca^2+^ mobilization was inhibited in the presence of 15‐HETE and 15‐HETrE. Collagen poorly activates platelets in the static conditions used to measure Ca^2+^ mobilization and integrin activation on a flow cytometer.^37^ Therefore, convulxin (CVX), a snake venom toxin, was used to stimulate GPVI for real‐time flow cytometer experiments. Pretreatment of platelets with 15‐HETrE or 15‐HETE (10 μM) resulted in a decrease in Ca^2+^ mobilization following stimulation with CVX compared to control‐treated platelets (Figure 4A). Since Ca^2+^ mobilization is required for activation of the integrin αIIbβ3, 15‐HETrE and 15‐HETE were assessed for their ability to attenuate αIIbβ3 activation. 15‐HETrE‐ or 15‐HETE‐treated platelets were stimulated with CVX (2.5 ng/mL) and activation was measured using flow cytometry in the presence of PAC‐1, an antibody that recognizes the active conformation of αIIbβ3. Compared to DMSO, treatment of platelets with 15‐HETrE or 15‐HETE inhibited αIIbβ3 activation in CVX‐stimulated platelets (Figure 4B).

Since the activation of conventional isoforms of PKC is dependent on Ca^2+^,^38^ the ability of 15‐HETrE and 15‐HETE to inhibit PKC in platelets was tested. At low concentrations of collagen (0.5 μg/mL), 15‐HETrE‐ and 15‐HETE‐treated platelets showed a reduced level of PKC substrate phosphorylation compared to the control (Figure 4C). However, there was no difference in PKC activation between control‐ and oxylipin‐treated platelets at higher concentrations of collagen (5 μg/mL) (Figure 4C). Agonist‐dependent granule release was also assessed as a measurement of platelet activation including both dense and α‐granules. To evaluate whether 15‐HETrE or 15‐HETE affects dense granule secretion, platelets were stimulated with increasing concentrations of collagen in the presence of 15‐HETrE, 15‐HETE, or control (DMSO). Collagen‐stimulated platelets incubated with 15‐HETE or 15‐HETrE released less ATP, a marker of dense granule secretion, than platelets treated with DMSO (Figure 4D). To determine if 15‐HETrE or 15‐HETE inhibited α‐granule secretion, platelets were stimulated with collagen (5 μg/mL) in the presence of 15‐HETrE or 15‐HETE, and surface expression of P‐selectin was measured by flow cytometer.^39^ Platelets treated with 15‐HETrE or 15‐HETE had a decrease in P‐selectin surface expression compared to control‐treated platelets (Figure 4E).

### 3.8 15‐HETrE and 15‐HETE inhibit platelet activation via unique PPARs

**FIGURE 5:**
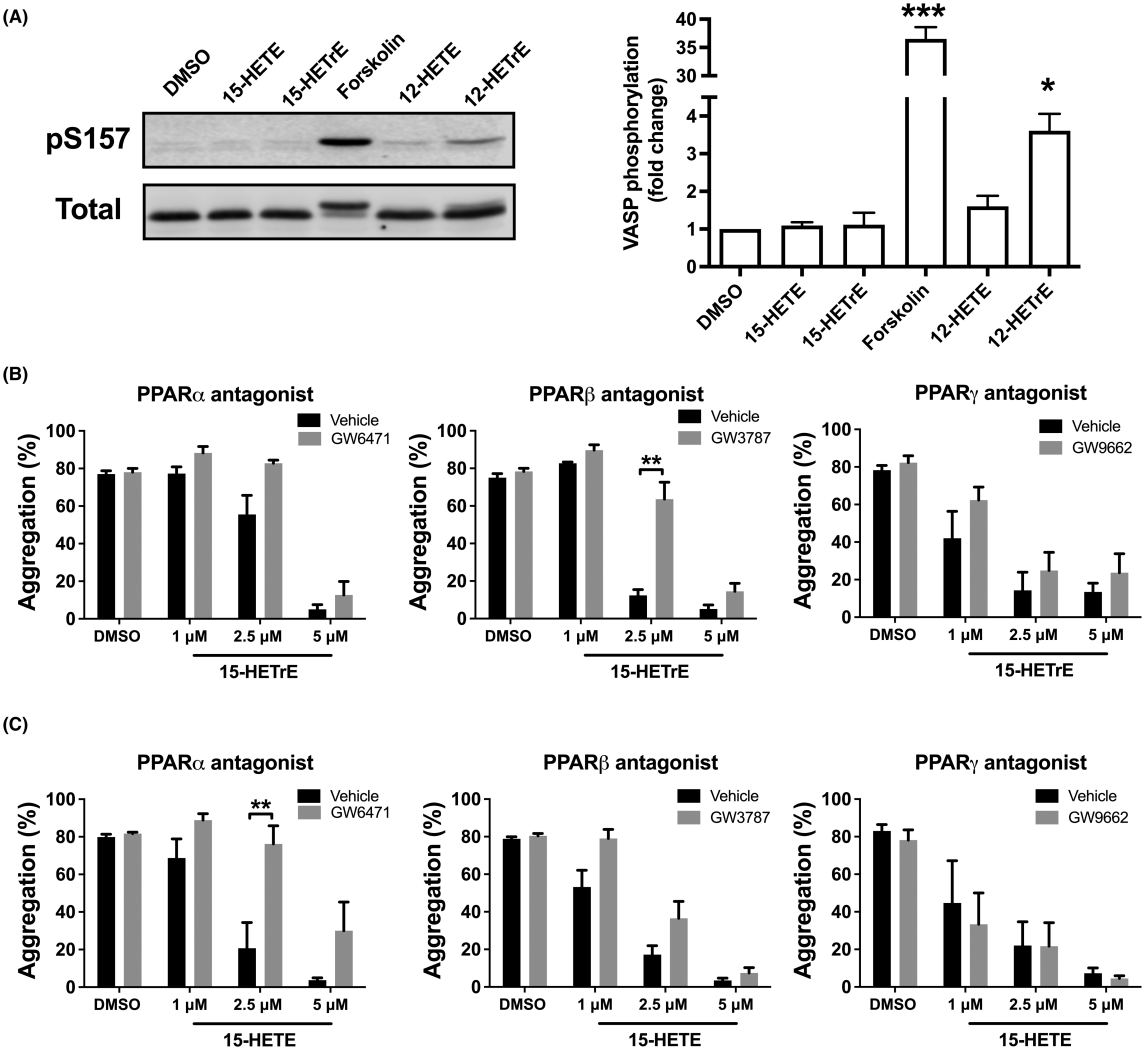
15‐HETE and 15‐HETrE inhibit platelet activation via distinct mechanisms. (A) The lysates of platelets (3 × 10^8^/mL) (*n* = 3‐4) treated with forskolin, a direct adenylyl cyclase agonist, oxylipins (10 μM) or DMSO were separated on a 10% SDS‐PAGE gel and western blots were performed with antibodies to phospho‐ and total VASP. (B) Platelets were incubated with PPARα (GW6471; 10 μM; *n* = 3‐6), PPARβ (GW3787; 10 μM; *n* = 3‐6), or PPARγ (GW9662; 10 μM; *n* = 3‐6) antagonist, prior to the treatment with 15‐LOX oxylipin, and then stimulated with collagen (0.25‐1 μg/mL). Data represent mean ± SEM. Two‐tailed paired *t* test. DMSO concentration corresponds to the residual DMSO in the 10 μM oxylipin sample.

Independent of direct effects on oxygenases, oxylipins have additionally been proposed to reduce platelet activation through either the initiation of Gα_s_‐coupled receptor signaling or stimulation of PPARs.^1^ The inhibitory effects of the Gα_s_ signaling pathway proceed through cAMP‐dependent PKA activation.^40^ In platelets, the major substrate of PKA is VASP serine 157 (S157).^41^ To determine if 15‐HETrE or 15‐HETE regulates platelet function in this manner, VASP phosphorylation was measured in platelets treated with oxylipins (10 μM) to assess their ability to initiate Gα_s_ signaling. VASP (S157) phosphorylation did not increase in platelets incubated with 15‐HETrE or 15‐HETE compared to either DMSO or 12‐HETE, a negative control (Figure 5A). As expected, platelets treated with either forskolin, a direct adenylyl cyclase agonist, or 12‐HETrE, a 12‐LOX oxylipin that signals through a Gα_s_‐coupled receptor,^20^ had enhanced VASP phosphorylation.

Platelets express all three PPAR isoforms (α, β, and γ) and activation of any of these isoforms inhibit platelet function through a non‐genomic mechanism.^42^, ^43^ Since 15‐HETrE and 15‐HETE have been shown to activate PPARs in other cell types,^44^, ^45^ we sought to determine if either 15‐HETrE or 15‐HETE inhibits platelet aggregation in a PPAR‐dependent manner in platelets. Platelets were incubated with the previously characterized inhibitors of PPARα (GW6471; 10 μM), PPARβ (GSK3787; 10 μM), or PPARγ (GW9662; 10 μM), prior to treatment with 15‐HETrE or 15‐HETE and subsequent collagen stimulation.^42^, ^46^, ^47^, ^48^ Inhibition of PPARβ, but not PPARα or PPARγ, reversed the antiplatelet effects of low concentrations of 15‐HETrE (2.5 μM) in collagen‐stimulated platelets (Figure 5B). In contrast, inhibition of PPARα, but not PPARβ or PPARγ, reversed the ability of low concentrations of 15‐HETE (2.5 μM) to inhibit collagen‐induced aggregation (Figure 5C). None of the PPAR antagonists tested were able to reverse the inhibitory effects of higher concentrations of 15‐HETrE or 15‐HETE (5 μM).

### 3.9 15‐LOX expression in human platelets

**FIGURE 6:**
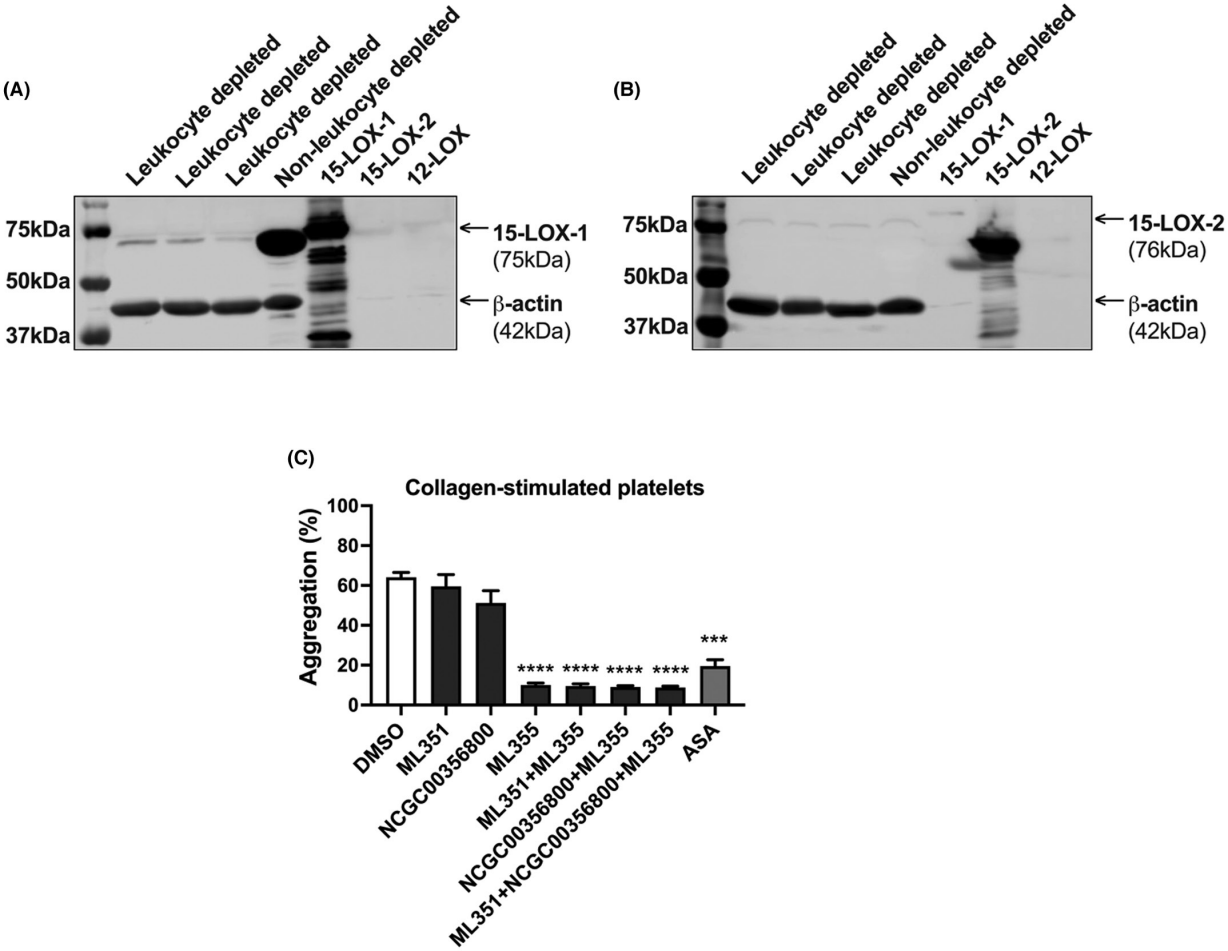
15‐LOX‐1 might be expressed in platelets, but not 15‐LOX‐2. The lysates of leukocyte‐depleted platelets (*n* = 3) and non‐depleted platelets (*n* = 1), 15‐LOX‐1, 15‐LOX‐2, and 12‐ LOX enzymes were separated on a 10% SDS‐PAGE gel and western blots were performed with antibodies to (A) 15‐LOX‐1 or (B) 15‐LOX‐2. An antibody to β‐actin was used as a loading control. Leukocyte‐depleted platelets were treated with 15‐LOX‐1 inhibitor (ML351, 10 μM), 15‐LOX‐2 inhibitor (NCGC00356800, 10 μM) or/and 12‐LOX inhibitor (ML355, 20 μM), or aspirin (ASA, 100 μM) for 10 min for ML351, NCGC00356800 or ML355 and 40 min for ASA, prior stimulation with 2 μg/mL of collagen and (C) platelet aggregation (*n* = 6) was assessed. Data represent mean ± SEM. One‐way ANOVA with Dunnett's multiple comparison post hoc test. *p < 0.05, **p < 0.01, ***p < 0.001, ***p < 0.0001. DMSO concentration corresponds to the residual DMSO in the 10 μM oxylipin sample.

In this study, we demonstrated that leukocyte‐depleted platelets treated with DGLA prior to agonist‐induced activation generated 15‐HETrE (Figure 1B); however, it remains unclear if 15‐LOX or COX‐1 produced the 15‐oxylipin. To assess whether 15‐LOX is expressed in platelets, both leukocyte‐depleted and non‐depleted platelets were probed with 15‐LOX‐1 or 15‐LOX‐2 antibodies. As a control, the purified 15‐LOX‐1, 15‐LOX‐2, and 12‐LOX enzymes^17^, ^49^, ^50^ were tested and as expected, the 15‐LOX‐1 and 15‐LOX‐2 antibodies selectively detected the corresponding 15‐LOX isozymes, but not the alternate 15‐LOX isozyme, nor the 12‐LOX enzyme. While 15‐LOX‐1 expression decreased in leukocyte‐depleted platelets compared to non‐depleted platelets (Figure 6A), the expression of 15‐LOX‐2 was absent either in the leukocyte‐depleted platelets and non‐depleted platelets (Figure 6B).

Leukocyte‐depleted platelets were treated with the 15‐LOX‐1 selective inhibitor, ML351,^51^ or the 15‐LOX‐2 selective inhibitor, NCGC00356800,^52^ and collagen‐induced platelet aggregation were assessed (Figure 6C). Platelets were also treated with the 12‐LOX selective inhibitor, ML355, and with aspirin (ASA), as controls. Agonist‐induced platelet aggregation was inhibited following treatment with ML355 or ASA. However, inhibition of only 15‐LOX‐1 or 15‐LOX‐2 did not affect collagen‐induced platelet aggregation.

## 4 DISCUSSION

Lipoxygenases (LOXs) are enzymes that catalyze the oxygenation of PUFAs, forming bioactive fatty acids (oxylipins).^53^, ^54^ LOXs (5‐, 12‐, and 15‐LOX) are expressed in a number of cells and they each produce oxylipins which regulate platelet activity, hemostasis, and thrombosis.^2^, ^6^ Regarding the expression of lipoxygenases in platelets, 5‐LOX is not expressed in these cells, whereas 12‐LOX is highly expressed^2^, ^55^ and its derived oxylipins are known to regulate platelet reactivity.^56^, ^57^ Whether 15‐LOX is expressed in platelets has been an ongoing question in the field. While the formation of 15‐oxylipins in platelets has already been shown, these studies are not in agreement regarding the role of 15‐LOX^8^ or COX‐1^9^, ^58^ in the formation of 15‐oxylipins in platelets.

In this study, we demonstrated that micromolar levels of 15‐HETE, 15‐HETrE, or 15‐HEPE attenuated collagen‐induced platelet aggregation. Although several studies reported the 15‐HETE and 15‐HEPE effects on platelet function,^10^, ^11^, ^23^, ^24^ the effects of 15‐HETrE on platelet activity are still not well understood. We have shown that 15‐HETrE at micromolar levels attenuated aggregation initiated by different agonists including collagen, thrombin, and ADP (Figure 2A). Due to the fact that the 15‐oxylipins negatively regulate platelet reactivity, the pharmacological inhibition of 15‐LOX‐1 or 15‐LOX‐2 in the platelet did not rescue agonist‐induced platelet aggregation (Figure 6C). However, inhibition of platelet aggregation was observed following treatment with ML355 or ASA, inhibitors for 12‐LOX and COX‐1, respectively. The mechanism underlying these effects is that 12‐HETE and thromboxane A_2_ (TXA_2_), the 12‐LOX‐derived and the COX‐1‐derived oxylipins from AA, respectively, have been demonstrated to potentiate platelet activation and aggregation.^59^, ^60^

The ability of 15‐oxylipins to inhibit platelet aggregation suggests that these oxylipins impinge on a common signaling event downstream of receptor activation in the platelet aggregation pathway. Since the 12‐LOX‐derived oxylipins from AA and DGLA, 12‐HETE and 12‐HETrE, respectively, play a critical role in regulating platelet reactivity,^14^, ^35^, ^60^ we focused on 15‐HETE and 15‐HETrE to investigate how the 15‐oxylipins were regulating platelet signaling following GPVI stimulation. We have shown that both 15‐HETE and 15‐HETrE inhibited the activation of common signaling events, including Ca^2+^ mobilization, and activation of integrin αIIbβ3 (Figure 4). Interestingly, while some 12‐LOX oxylipins such as 12‐HETrE have been previously shown by our group to inhibit platelet function through activation of the prostacyclin receptor on the surface of the platelet resulting in activation of Gα_s_, formation of cAMP, and activation of PKA,^14^ neither 15‐HETrE nor 15‐HETE resulted in VASP phosphorylation by PKA, suggesting that these oxylipins inhibit platelet function in a prostacyclin receptor‐independent manner (Figure 5).

While several key biochemical steps such as calcium mobilization, PKC activation, and integrin activation were shown in the current study to be similarly regulated by 15‐HETE and 15‐HETrE, the proximal regulatory steps preceding these central biochemical regulators were identified as unique to each of the metabolites studied. Previously, our group showed that docosapentaenoic acid (DPA) ω‐6‐derived oxylipins activate PPARs in the platelet^57^ and others demonstrated that 15‐oxylipins activate PPARs in other cells.^44^, ^61^ In this study, we have shown for the first time that the 15‐oxylipins, 15‐HETrE and 15‐HETE, signal at least partially through activation of PPARs in the platelet. While 15‐HETrE was found to be an agonist for PPARβ, 15‐HETE appears to function through the activation of PPARα (Figure 5). PPAR antagonists reversed the inhibitory effects of lower concentrations of 15‐HETE and 15‐HETrE; however, PPAR inhibitors could not reverse higher concentrations of these oxylipins. Hence, it is reasonable to suggest that 15‐HETE and 15‐HETrE function partially through PPARs, but that at higher concentrations they may signal through other compensatory signaling pathways in the platelet independent of PPAR signaling.

A number of monohydroxylated oxylipins have antiplatelet activity; however, how the structure of these oxylipins regulates their mechanism of action remains poorly understood.^2^ Independent discoveries demonstrated that 19(S)‐hydroxyeicosatetraenoic acid (19‐HETE)^62^ and 12‐HETrE^14^ both inhibit platelet activation by signaling through the prostacyclin receptor, but the activity of other oxylipins warranted a further investigation into the structure‐activity relationship of antiplatelet monohydroxylated oxylipins. A number of physical attributes of monohydroxylated oxylipins have been shown to influence their functionality, such as carbon length, double bond configuration, and position/stereochemistry of oxygenation. HETEs and HETrEs both have 20 carbon backbones and double bonds at the 8, 10, and 14 carbons, but HETEs contain an additional double bond at the 5th carbon. 12‐HETE and 12‐HETrE have been shown to have opposite effects on platelet activity, suggesting that the double bond configuration may be a major contributor to monohydroxylated oxylipin function.^20^ Notably, in contrast to the opposite effects observed with 12‐HETE and 12‐HETrE, this study found that 15‐HETE and 15‐HETrE have similar but unique functionality. These data suggest that the difference in a single double bond does not change the overall inhibitory effect in platelet function of these 15‐oxylipins, as observed with 12‐HETE and 12‐HETrE, but rather shifts the isoform of PPAR that is activated.

Oxylipin inhibition of platelet function through negative feedback on the production of pro‐aggregatory oxylipins has been previously shown.^24^ In agreement with previous studies,^10^, ^22^, ^33^ we observe that 15‐oxylipins were shown in the current study to partially inhibit 12‐HETE formation, which helps explain the inability of PPAR inhibitors to fully reverse 15‐oxylipins' antiplatelet effects. Although the in vitro data suggest that the 15‐oxylipins do not directly inhibit recombinant 12‐LOX, we have observed that in ex vivo experiments using human platelets, the 15‐oxylipins showed a differential ability to inhibit 12‐LOX, with platelets treated with 15‐HETE, 15‐HETrE or 15‐HEPE having a 90%, 40%, and 89% decrease in 12‐LOX product formation, respectively (Figure 3). However, the COX‐derived product of AA, TXB_2_, was not decreased in platelets treated with either 15‐HETE, 15‐HETrE, or 15‐HEPE, which suggests that these 15‐oxylipins are selectively lowering 12‐LOX activity, without lowering the availability of the substrate, AA. It should be noted that while 15‐oxylipins are allosteric regulators of 12‐LOX reactivity, they are poor substrates of 12‐LOX both in vitro and ex vivo, indicating that 15‐oxylipins are the primary biomolecules in the platelet.

Previously, our group has demonstrated that treatment with DGLA increased levels of the 12‐LOX‐derived oxylipin, 12‐HETrE, in platelets.^20^ In accordance with previous findings,^8^, ^9^ we observe that 15‐HETrE was detected in the releasate of leukocyte‐depleted platelets treated with DGLA (Figure 1B), suggesting that platelets have the ability to generate 15‐oxylipins, but the expression of 15‐LOX in platelets remains unclear. Mammalian tissues have two forms of 15‐LOX isoforms, reticulocyte 15‐LOX (15‐LOX‐1, gene ALOX15) and epithelial 15‐LOX‐2 (15‐LOX‐2, gene ALOX15B), with the tissue distribution of 15‐LOX‐2 being more limited when compared to that of 15‐LOX‐1.^61^, ^63^ While 15‐LOX‐2 is predominantly found in the skin, prostate, lung, and cornea, 15‐LOX‐1 is expressed in eosinophils, leukocytes, reticulocytes, macrophages, dendritic, and epithelial cells.^64^, ^65^ In agreement with those observations, antibodies for 15‐LOX‐1 or 15‐LOX‐2 demonstrated that platelets may express low levels of 15‐LOX‐1, but do not express 15‐LOX‐2 (Figure 6).

**FIGURE 7:**
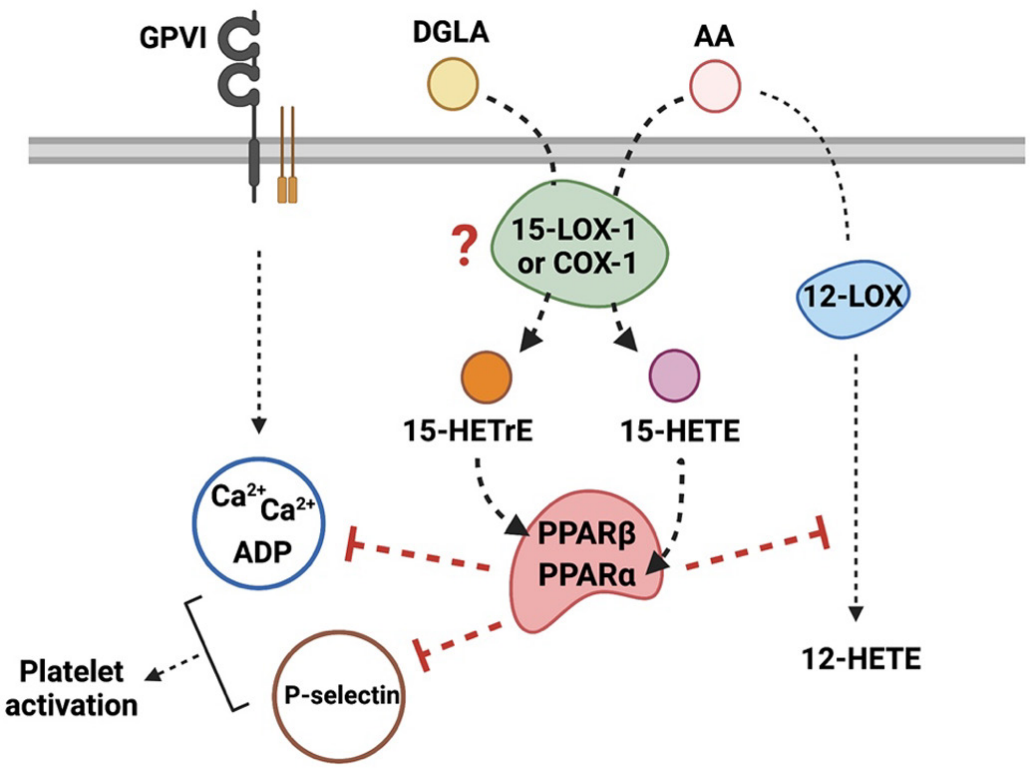
Schematic overview of the mechanism underlying the inhibitory effect of 15‐ oxylipins on platelet reactivity. In platelets, 15‐LOX‐1 or COX‐1 might metabolize free DGLA and AA into 15‐HETrE and 15‐HETE, respectively. Both oxylipins act partially through activation of PPARs, impinging intracellular signaling and inhibiting 12‐LOX activity, which leads to the inhibition of platelet activation in response to collagen.

Our study has certain limitations. Although we demonstrated that platelets produce 15‐HETrE in vitro and the 15‐oxylipins attenuate platelet reactivity, we were not able to determine whether 15‐LOX is expressed in the platelets and required for the formation of the 15‐oxylipins in platelets. This study suggests that 15‐LOX‐1 might be expressed in platelets at low levels. However, based on the significant difference observed in the enzyme's expression between leukocyte‐depleted and non‐depleted platelets, it is reasonable to consider that if platelets have 15‐LOX‐1, the expression is at a low concentration. It is also possible that the 15‐oxylipins might be formed through a 15‐LOX‐independent pathway (Figure 7). Indeed, previous studies have suggested that 15‐oxylipins are produced in a COX‐dependent manner in platelets^9^, ^58^, ^66^ and demonstrated that recombinant COX has the ability to metabolize AA into 15(S)‐HETE in vitro.^67^ Hence, in future studies, mass spectrometry analysis using pharmacological inhibition of 15‐LOX or COX‐1 in platelets could help to determine which oxygenase might be involved in the formation of the 15‐oxylipins. Regardless of the source of the 15‐oxylipins, this study suggests that platelets not only form 15‐oxylipins but that they have antiplatelet effects. Hence, it is possible that under physiologic conditions, 15‐oxylipins may play an important regulatory role in the onset and stability of the blood clot in the blood vessel. 15‐oxylipins could prevent newly recruited platelets from becoming active at the site of injury, which might regulate the formation of the clot and further attenuate or reduce the thrombotic risk. Furthermore, based on our findings and the fact that 15‐LOX‐1 is highly expressed in leukocytes,^2^ it is reasonable to consider that in whole blood, platelet reactivity might be partially regulated by a transcellular mechanism between platelets and leukocytes through the formation of 15‐oxylipins. Therefore, this leukocyte‐platelet interaction could regulate clot formation and thus have clinical implications in atherothrombotic diseases through inhibition of platelet activity and thrombosis.

## AUTHOR CONTRIBUTIONS

Yamaguchi, van Hoorebeke, Tourdot, Perry, Holman, and Holinstat: Participated in research design. Yamaguchi, van Hoorebeke, Tourdot, Perry, Lee, Rhoads, Rickenberg, Green, Sorrentino, Yeung, and Freedman: Conducted experiments. Yamaguchi, van Hoorebeke, Tourdot, Perry, Holman, and Holinstat: Performed data analysis. : Yamaguchi, van Hoorebeke, Tourdot, Perry, Holman, and Holinstat: Wrote or contributed to the writing of the manuscript.

## FUNDING INFORMATION

This study was supported by NIH grants R35 GM131835 (MH), R01 AG047986 (MH & TH), F31HL129481 (JY), K99 HL136784 (BT), T32 HL007853 (AY), and UL1 TR002240 (MH and AY).

## CONFLICT OF INTEREST STATEMENT

Dr. Holinstat is a consultant and equity holder and consultant for Veralox therapeutics and Cereno Scientific. All other authors declare no competing interests for the work reported in this manuscript.

## ETHICS STATEMENT

All subjects participated voluntarily and received a small compensation. The participants provided their written informed consent to participated in this study. The Declaration of Helsinki was adequately addressed, and the study was approved by the University of Michigan Institutional Review Board.

## Data Availability

The data that support the findings of this study are available from the corresponding author upon reasonable request.

## References

[1] Tourdot BE , Ahmed I , Holinstat M . The emerging role of oxylipins in thrombosis and diabetes. Front Pharmacol. 2014;4:176. doi:10.3389/fphar.2013.00176 24432004 PMC3882718

[2] Yeung J , Hawley M , Holinstat M . The expansive role of oxylipins on platelet biology. J Mol Med (Berl). 2017;95(6):575‐588. doi:10.1007/s00109-017-1542-4 28528513 PMC5547901

[3] Adili R , Hawley M , Holinstat M . Regulation of platelet function and thrombosis by omega‐3 and omega‐6 polyunsaturated fatty acids. Prostaglandins Other Lipid Mediat. 2018;139:10‐18. doi:10.1016/j.prostaglandins.2018.09.005 30266534 PMC6242736

[4] Schrör K . Aspirin and platelets: the antiplatelet action of aspirin and its role in thrombosis treatment and prophylaxis. Semin Thromb Hemost. 1997;23(4):349‐356. doi:10.1055/s-2007-996108 9263351

[5] Adili R , Tourdot BE , Mast K , et al. First selective 12‐ LOX inhibitor, ML355, impairs thrombus formation and vessel occlusion In vivo with minimal effects on hemostasis. Arterioscler Thromb Vasc Biol. 2017;37(10):1828‐1839. doi:10.1161/atvbaha.117.309868 28775075 PMC5620123

[6] Yeung J , Holinstat M . 12‐lipoxygenase: a potential target for novel anti‐platelet therapeutics. Cardiovasc Hematol Agents Med Chem. 2011;9(3):154‐164. doi:10.2174/187152511797037619 21838667 PMC3171607

[7] Kim HY , Karanian JW , Salem N Jr . Formation of 15‐lipoxygenase product from docosahexaenoic acid (22:6w3) by human platelets. Prostaglandins. 1990;40(5):539‐549. doi:10.1016/0090-6980(90)90115-c 2147774

[8] Wong PY , Westlund P , Hamberg M , Granström E , Chao PH , Samuelsson B . 15‐Lipoxygenase in human platelets. J Biol Chem. 1985;260(16):9162‐9165. 3926763

[9] Guichardant M , Naltachayan‐Durbin S , Lagarde M . Occurrence of the 15‐hydroxy derivative of dihomogammalinolenic acid in human platelets and its biological effect. Biochim Biophys Acta. 1988;962(1):149‐154. doi:10.1016/0005-2760(88)90106-3 2843240

[10] Vanderhoek JY , Schoene NW , Pham PP . Inhibitory potencies of fish oil hydroxy fatty acids on cellular lipoxygenases and platelet aggregation. Biochem Pharmacol. 1991;42(4):959‐962. doi:10.1016/0006-2952(91)90062-a 1831021

[11] Setty BN , Stuart MJ . 15‐Hydroxy‐5,8,11,13‐eicosatetraenoic acid inhibits human vascular cyclooxygenase. Potential role in diabetic vascular disease. J Clin Invest. 1986;77(1):202‐211. doi:10.1172/jci112277 3080473 PMC423328

[12] Vijil C , Hermansson C , Jeppsson A , Bergström G , Hultén LM . Arachidonate 15‐lipoxygenase enzyme products increase platelet aggregation and thrombin generation. PLoS One. 2014;9(2):e88546. doi:10.1371/journal.pone.0088546 24533104 PMC3922896

[13] Kozak KR , Gupta RA , Moody JS , et al. 15‐lipoxygenase metabolism of 2‐arachidonylglycerol. Generation of a peroxisome proliferator‐activated receptor alpha agonist. J Biol Chem. 2002;277(26):23278‐23286. doi:10.1074/jbc.M201084200 11956198

[14] Tourdot BE , Adili R , Isingizwe ZR , et al. 12‐HETrE inhibits platelet reactivity and thrombosis in part through the prostacyclin receptor. Blood Adv. 2017;1(15):1124‐1131. doi:10.1182/bloodadvances.2017006155 29296755 PMC5728320

[15] Smyrniotis CJ , Barbour SR , Xia Z , Hixon MS , Holman TR . ATP allosterically activates the human 5‐lipoxygenase molecular mechanism of arachidonic acid and 5(S)‐ hydroperoxy‐6(E),8(Z),11(Z),14(Z)‐eicosatetraenoic acid. Biochemistry. 2014;53(27):4407‐4419. doi:10.1021/bi401621d 24893149 PMC4215895

[16] Wecksler AT , Kenyon V , Garcia NK , Deschamps JD , van der Donk WA , Holman TR . Kinetic and structural investigations of the allosteric site in human epithelial 15‐lipoxygenase‐2. Biochemistry. 2009;48(36):8721‐8730. doi:10.1021/bi9009242 19645454 PMC2746553

[17] Amagata T , Whitman S , Johnson TA , et al. Exploring sponge‐derived terpenoids for their potency and selectivity against 12‐human, 15‐human, and 15‐soybean lipoxygenases. J Nat Prod. 2003;66(2):230‐235. doi:10.1021/np020462l 12608855

[18] Butovich IA . A one‐step method of 10,17‐dihydro(pero)xydocosahexa‐ 4Z,7Z,11 E,13Z,15 E,19Z‐enoic acid synthesis by soybean lipoxygenase. J Lipid Res. 2006;47(4):854‐863. doi:10.1194/jlr.D500042-JLR200 16391324

[19] Serhan CN , Dalli J , Karamnov S , et al. Macrophage proresolving mediator maresin 1 stimulates tissue regeneration and controls pain. Faseb J. 2012;26(4):1755‐1765. doi:10.1096/fj.11-201442 22253477 PMC3316905

[20] Yeung J , Tourdot BE , Adili R , et al. 12(S)‐HETrE, a 12‐lipoxygenase oxylipin of dihomo‐γ‐Linolenic acid, inhibits thrombosis via gas signaling in platelets. Arterioscler Thromb Vasc Biol. 2016;36(10):2068‐2077. doi:10.1161/atvbaha.116.308050 27470510 PMC5488693

[21] Paul BZ , Jin J , Kunapuli SP . Preparation of mRNA and cDNA libraries from platelets and megakaryocytes. Methods Mol Biol. 2004;273:435‐454. doi:10.1385/1-59259-783-1:435 15308817

[22] Vanderhoek JY , Bailey JM . Activation of a 15‐lipoxygenase/leukotriene pathway in human polymorphonuclear leukocytes by the anti‐inflammatory agent ibuprofen. J Biol Chem. 1984;259(11):6752‐6756. 6427221

[23] Vedelago HR , Mahadevappa VG . Differential effects of 15‐HPETE on arachidonic acid metabolism in collagen‐stimulated human platelets. Biochem Biophys Res Commun. 1988;150(1):177‐184. doi:10.1016/0006-291x(88)90502-5 3122753

[24] Vericel E , Lagarde M . 15‐Hydroperoxyeicosatetraenoic acid inhibits human platelet aggregation. Lipids. 1980;15(6):472‐474. doi:10.1007/bf02534075 7401944

[25] Dadaian M , Westlund P . Albumin modifies the metabolism of hydroxyeicosatetraenoic acids via 12‐lipoxygenase in human platelets. J Lipid Res. 1999;40(5):940‐947. 10224163

[26] Bild GS , Bhat SG , Axelrod B , Iatridis PG . Inhibition of aggregation of human platelets by 8,15‐dihydroperoxides of 5,9,11,13‐eicosatetraenoic and 9,11,13‐eicosatrienoic acids. Prostaglandins. 1978;16(5):795‐801. doi:10.1016/00906980(78)90012-6

[27] Kutzner L , Goloshchapova K , Rund KM , et al. Human lipoxygenase isoforms form complex patterns of double and triple oxygenated compounds from eicosapentaenoic acid. Biochim Biophys Acta Mol Cell Biol Lipids. 2020;1865(12):158806. doi:10.1016/j.bbalip.2020.158806 32841762

[28] Maas RL , Brash AR . Evidence for a lipoxygenase mechanism in the biosynthesis of epoxide and dihydroxy leukotrienes from 15(S)‐hydroperoxyicosatetraenoic acid by human platelets and porcine leukocytes. Proc Natl Acad Sci USA. 1983;80(10):2884‐2888. doi:10.1073/pnas.80.10.2884 6304687 PMC393937

[29] Freedman C , Tran A , Tourdot BE , et al. Biosynthesis of the Maresin intermediate, 13 S,14 S‐epoxy‐ DHA, by human 15‐lipoxygenase and 12‐lipoxygenase and its regulation through negative allosteric modulators. Biochemistry. 2020;59(19):1832‐1844. doi:10.1021/acs.biochem.0c00233 32324389 PMC7729281

[30] Perry SC , Kalyanaraman C , Tourdot BE , et al. 15‐Lipoxygenase‐1 biosynthesis of 7 S,14 S‐diHDHA implicates 15‐lipoxygenase‐2 in biosynthesis of resolvin D5. J Lipid Res. 2020;61(7):1087‐1103. doi:10.1194/jlr.RA120000777 32404334 PMC7328043

[31] Tsai WC , Kalyanaraman C , Yamaguchi A , Holinstat M , Jacobson MP , Holman TR . In vitro biosynthetic pathway investigations of Neuroprotectin D1 (NPD1) and Protectin DX (PDX) by human 12‐lipoxygenase, 15‐Lipoxygenase‐1, and 15‐Lipoxygenase‐2. Biochemistry. 2021;60(22):1741‐1754. doi:10.1021/acs.biochem.0c00931 34029049 PMC9007043

[32] Spector AA , Gordon JA , Moore SA . Hydroxyeicosatetraenoic acids (HETEs). Prog Lipid Res. 1988;27(4):271‐323. doi:10.1016/0163-7827(88)90009-4 3076240

[33] Mitchell PD , Hallam C , Hemsley PE , Lord GH , Wilkinson D . Inhibition of platelet 12‐lipoxygenase by hydroxy‐fatty acids. Biochem Soc Trans. 1984;12(5):839‐841. doi:10.1042/bst0120839

[34] Holinstat M , Boutaud O , Apopa PL , et al. Protease‐activated receptor signaling in platelets activates cytosolic phospholipase A2α differently for cyclooxygenase‐1 and 12‐lipoxygenase catalysis. Arterioscler Thromb Vasc Biol. 2011;31(2):435‐442. doi:10.1161/atvbaha.110.219527 21127289 PMC3035574

[35] Sekiya F , Takagi J , Usui T , et al. 12 S‐hydroxyeicosatetraenoic acid plays a central role in the regulation of platelet activation. Biochem Biophys Res Commun. 1991;179(1):345‐351. doi:10.1016/0006-291x(91)91376-n 1652954

[36] Ikei KN , Yeung J , Apopa PL , et al. Investigations of human platelet‐type 12‐lipoxygenase: role of lipoxygenase products in platelet activation. J Lipid Res. 2012;53(12):2546‐2559. doi:10.1194/jlr.M026385 22984144 PMC3494251

[37] Moers A , Wettschureck N , Grüner S , Nieswandt B , Offermanns S . Unresponsiveness of platelets lacking both Galpha(q) and Galpha(13). Implications for collagen‐induced platelet activation. J Biol Chem. 2004;279(44):45354‐45359. doi:10.1074/jbc.M408962200 15326177

[38] Harper MT , Poole AW . Diverse functions of protein kinase C isoforms in platelet activation and thrombus formation. J Thromb Haemost. 2010;8(3):454‐462. doi:10.1111/j.1538-7836.2009.03722.x 20002545

[39] Furie B , Furie BC , Flaumenhaft R . A journey with platelet P‐selectin: the molecular basis of granule secretion, signalling and cell adhesion. Thromb Haemost. 2001;86(1):214‐221. 11487009

[40] Smolenski A . Novel roles of cAMP/cGMP‐dependent signaling in platelets. J Thromb Haemost. 2012;10(2):167‐176. doi:10.1111/j.1538-7836.2011.04576.x 22136590

[41] Butt E , Abel K , Krieger M , et al. cAMP‐ and cGMP‐dependent protein kinase phosphorylation sites of the focal adhesion vasodilator‐ stimulated phosphoprotein (VASP) in vitro and in intact human platelets. J Biol Chem. 1994;269(20):14509‐14517. 8182057

[42] Ali FY , Davidson SJ , Moraes LA , et al. Role of nuclear receptor signaling in platelets: antithrombotic effects of PPARbeta. FASEB J. 2006;20(2):326‐328. doi:10.1096/fj.05-4395fje 16368717

[43] Lannan KL , Sahler J , Kim N , et al. Breaking the mold: transcription factors in the anucleate platelet and platelet‐derived microparticles. Front Immunol. 2015;6:48. doi:10.3389/fimmu.2015.00048 25762994 PMC4327621

[44] Naruhn S , Meissner W , Adhikary T , et al. 15‐hydroxyeicosatetraenoic acid is a preferential peroxisome proliferator‐activated receptor beta/delta agonist. Mol Pharmacol. 2010;77(2):171‐184. doi:10.1124/mol.109.060541 19903832

[45] Thuillier P , Brash AR , Kehrer JP , et al. Inhibition of peroxisome proliferator‐activated receptor (PPAR)‐mediated keratinocyte differentiation by lipoxygenase inhibitors. Biochem J. 2002;366(Pt 3):901‐910. doi:10.1042/bj20020377 12069687 PMC1222830

[46] Ali FY , Armstrong PC , Dhanji AR , et al. Antiplatelet actions of statins and fibrates are mediated by PPARs. Arterioscler Thromb Vasc Biol. 2009;29(5):706‐711. doi:10.1161/atvbaha.108.183160 19150877

[47] Du H , Hu H , Zheng H , Hao J , Yang J , Cui W . Effects of peroxisome proliferator‐ activated receptor γ in simvastatin antiplatelet activity: influences on cAMP and mitogen‐ activated protein kinases. Thromb Res. 2014;134(1):111‐120. doi:10.1016/j.thromres.2014.05.005 24856644

[48] Moraes LA , Spyridon M , Kaiser WJ , et al. Non‐genomic effects of PPARgamma ligands: inhibition of GPVI‐stimulated platelet activation. J Thromb Haemost. 2010;8(3):577‐587. doi:10.1111/j.1538-7836.2009.03732.x 20040043 PMC3298645

[49] Joshi N , Hoobler EK , Perry S , Diaz G , Fox B , Holman TR . Kinetic and structural investigations into the allosteric and pH effect on the substrate specificity of human epithelial 15‐lipoxygenase‐2. Biochemistry. 2013;52(45):8026‐8035. doi:10.1021/bi4010649 24171444 PMC3866584

[50] Robinson SJ , Hoobler EK , Riener M , et al. Using enzyme assays to evaluate the structure and bioactivity of sponge‐derived meroterpenes. J Nat Prod. 2009;72(10):1857‐1863. doi:10.1021/np900465e 19848434 PMC2996101

[51] Rai, G. , Joshi, N. , Perry, S. , et al. Discovery of ML351, a Potent and Selective Inhibitor of Human 15‐Lipoxygenase‐1. In Probe Reports from the NIH Molecular Libraries Program. Bethesda (MD): National Center for Biotechnology Information (US). 2010. 24672829

[52] Jameson JB 2nd , Kantz A , Schultz L , et al. A high throughput screen identifies potent and selective inhibitors to human epithelial 15‐lipoxygenase‐2. PLoS One. 2014;9(8):e104094. doi:10.1371/journal.pone.0104094 25111178 PMC4128814

[53] Kuhn H , Walther M , Kuban RJ . Mammalian arachidonate 15‐lipoxygenases structure, function, and biological implications. Prostaglandins Other Lipid Mediat. 2002;68‐69:263‐290. doi:10.1016/s0090-6980(02)00035-7 12432923

[54] Orafaie A , Mousavian M , Orafai H , Sadeghian H . An overview of lipoxygenase inhibitors with approach of in vivo studies. Prostaglandins Other Lipid Mediat. 2020;148:106411. doi:10.1016/j.prostaglandins.2020.106411 31953016

[55] Funk CD , Furci L , FitzGerald GA . Molecular cloning, primary structure, and expression of the human platelet/erythroleukemia cell 12‐lipoxygenase. Proc Natl Acad Sci U S A. 1990;87(15):5638‐5642. doi:10.1073/pnas.87.15.5638 2377602 PMC54382

[56] Yamaguchi A , Stanger L , Freedman CJ , et al. DHA 12‐LOX‐derived oxylipins regulate platelet activation and thrombus formation through a PKA‐dependent signaling pathway. J Thromb Haemost. 2021;19(3):839‐851. doi:10.1111/jth.15184 33222370 PMC7925359

[57] Yeung J , Adili R , Yamaguchi A , et al. Omega‐6 DPA and its 12‐lipoxygenase‐oxidized lipids regulate platelet reactivity in a nongenomic PPARα‐dependent manner. Blood Adv. 2020;4(18):4522‐4537. doi:10.1182/bloodadvances.2020002493 32946570 PMC7509882

[58] Rauzi F , Kirkby NS , Edin ML , et al. Aspirin inhibits the production of proangiogenic 15(S)‐HETE by platelet cyclooxygenase‐1. FASEB J. 2016;30(12):4256‐4266. doi:10.1096/fj.201600530R 27633788 PMC5102123

[59] Maskrey BH , Rushworth GF , Law MH , et al. 12‐hydroxyeicosatetraenoic acid is associated with variability in aspirin‐induced platelet inhibition. J Inflamm (Lond). 2014;11(1):33. doi:10.1186/s12950-014-0033-4 25349537 PMC4209229

[60] Porro B , Songia P , Squellerio I , Tremoli E , Cavalca V . Analysis, physiological and clinical significance of 12‐HETE: a neglected platelet‐derived 12‐lipoxygenase product. J Chromatogr B Analyt Technol Biomed Life Sci. 2014;964:26‐40. doi:10.1016/j.jchromb.2014.03.015 24685839

[61] Shappell SB , Keeney DS , Zhang J , Page R , Olson SJ , Brash AR . 15‐Lipoxygenase‐2 expression in benign and neoplastic sebaceous glands and other cutaneous adnexa. J Invest Dermatol. 2001;117(1):36‐43. doi:10.1046/j.1523-1747.2001.01378.x 11442747

[62] Tunaru S , Chennupati R , Nüsing RM , Offermanns S . Arachidonic acid metabolite 19(S)‐HETE induces Vasorelaxation and platelet inhibition by activating prostacyclin (IP) receptor. PLoS One. 2016;11(9):e0163633. doi:10.1371/journal.pone.0163633 27662627 PMC5035018

[63] Brash AR , Boeglin WE , Chang MS . Discovery of a second 15 S‐lipoxygenase in humans. Proc Natl Acad Sci USA. 1997;94(12):6148‐6152. doi:10.1073/pnas.94.12.6148 9177185 PMC21017

[64] Ivanov I , Kuhn H , Heydeck D . Structural and functional biology of arachidonic acid 15‐lipoxygenase‐1 (ALOX15). Gene. 2015;573(1):1‐32. doi:10.1016/j.gene.2015.07.073 26216303 PMC6728142

[65] Jiang WG , Watkins G , Douglas‐Jones A , Mansel RE . Reduction of isoforms of 15‐lipoxygenase (15‐LOX)‐1 and 15‐LOX‐2 in human breast cancer. Prostaglandins Leukot Essent Fatty Acids. 2006;74(4):235‐245. doi:10.1016/j.plefa.2006.01.009 16556493

[66] Turnbull RE , Sander KN , Turnbull J , Barrett DA , Goodall AH . Profiling oxylipins released from human platelets activated through the GPVI collagen receptor. Prostaglandins Other Lipid Mediat. 2022;158:106607. doi:10.1016/j.prostaglandins.2021.106607 34942378

[67] Thuresson ED , Lakkides KM , Smith WL . Different catalytically competent arrangements of arachidonic acid within the cyclooxygenase active site of prostaglandin endoperoxide H synthase‐1 lead to the formation of different oxygenated products. J Biol Chem. 2000;275(12):8501‐8507. doi:10.1074/jbc.275.12.8501 10722687

